# Mechanism Underlying the Color Differences in Apples Based on Metabolomic and Transcriptomic Analyses

**DOI:** 10.3390/ijms27177954

**Published:** 2026-09-07

**Authors:** Haidong Bu, Junling Qiao, Yige Jiang, Wenquan Yu, Hui Yuan

**Affiliations:** 1 Key Laboratory of Cold Region Fruit Breeding and Cultivation (Heilongjiang Province), Mudanjiang Branch of Heilongjiang Academy of Agricultural Sciences, Mudanjiang 157000, China; buhaidong11@126.com; 2 Key Laboratory of Fruit Postharvest Biology (Liaoning Province), College of Horticulture, Shenyang Agricultural University, Shenyang 110866, China

**Keywords:** apple, red peel, cyanidin-3-O-malonylglucoside, *MdDFR*, bud sport

## Abstract

Apple peel color is a key determinant of fruit quality and consumer preference. To elucidate the molecular basis underlying the peel color difference between ‘Jinhong’ (JH) apples and ‘Hongjinhong’ apples (HJH, a deep red bud sport of JH apples), we integrated physiological assays, widely targeted metabolomics, transcriptomics, and quantitative real-time PCR (qRT-PCR) to analyze peel pigment contents, metabolite profiles, and gene expression dynamics during fruit development. The deep red phenotype of HJH peel was primarily attributed to enhanced anthocyanin accumulation rather than alterations in chlorophyll content. Metabolomics identified 418 differentially accumulated metabolites. Among these, three cyanidin-type anthocyanins––cyanidin-3-O-(6″-O-malonyl) glucoside, cyanidin-3-O-(2″-O-xylosyl) galactoside, and cyanidin-3-O-glucoside––were significantly upregulated in HJH peel, serving as the key pigments responsible for its deep red coloration. Transcriptomics revealed 533 differentially expressed genes. Combined with qRT-PCR validation, the anthocyanin biosynthetic gene *MdDFR* (dihydroflavonol reductase) exhibited consistently high expression during critical developmental stages of HJH fruit, closely mirroring the anthocyanin accumulation pattern. Collectively, the upregulation of *MdDFR* drives the specific accumulation of cyanidin-type anthocyanins, representing the core molecular event underlying the deep red peel color of HJH apples. This study provides novel insights into the regulatory network of apple coloration and offers candidate genes for the genetic improvement of fruit quality.

## 1. Introduction

Apples (*Malus domestica* Borkh.) are one of the most economically important temperate fruit crops worldwide. Apple color is a critical appearance quality trait that directly influences consumer acceptance and commercial value [1,2,3]. Red-skinned cultivars are especially favored, as their vivid coloration derives largely from anthocyanins, a class of flavonoid secondary metabolites that not only enhance visual appeal, but also confer substantial nutritional and pharmacological benefits to humans [4,5,6,7]. These compounds exhibit a broad spectrum of bioactivities, including antioxidant, anti-inflammatory, anti-allergic, cardiovascular-protective, and night blindness-preventive effects, supporting the concept that a higher dietary intake of anthocyanin-rich fruits can contribute to disease prevention [8,9,10,11,12]. Among the various anthocyanin types identified in fruits, cyanidin derivatives predominate, and anthocyanin concentration is generally positively correlated with the intensity of fruit coloration [11,13,14,15]. In apple peel, the principal anthocyanins are cyanidin-3-glucoside, cyanidin-3-xyloside, cyanidin-3-rutinoside, cyanidin-3-arabinoside, and cyanidin-3-galactoside [16,17]. Notably, both the content and the composition of anthocyanins vary dynamically during fruit development and differ considerably among varieties [18,19].

Anthocyanin biosynthesis is governed by structural genes, which are conserved across different plant species and encode enzymes in the anthocyanin biosynthesis pathway. These structural genes comprise two key groups: the early biosynthetic genes and the late biosynthetic genes. In the fruit anthocyanin biosynthesis pathway, the early genes include *PAL* (phenylalanine ammonia-lyase), *CHS* (chalcone synthase), *CHI* (chalcone isomerase), and *F3H* (flavanone-3-hydroxylase), which are also involved in flavonol biosynthesis; their expression changes during fruit development [20]. In the fruit anthocyanin biosynthesis pathway, the late biosynthetic genes include *DFR* (dihydroflavonol reductase), *ANS* (anthocyanidin synthase), and *UFGT* (UDP-glucose: flavonoid-3-O-glucosyltransferase), which generally exhibit higher expression in vividly colored fruits [20]. Studies have shown that, among various light wavelengths, blue light and ultraviolet light effectively promote anthocyanin accumulation in detached, bagged apples by enhancing PAL activity [21]. During apple development, several genes, including *UFGT*, *CHS*, and *DFR*, are coordinately expressed to promote anthocyanin accumulation [22]. In peaches, *CHS* and *DFR* have been identified as key regulatory genes in anthocyanin biosynthesis [23]. Studies have shown that the expression level of *CHI* directly influences anthocyanin content in apples [24]. In snapdragons, mutation of *F3H* blocks anthocyanin biosynthesis, resulting in white flowers [25]. Silencing the *F3H* gene in carnations shifts flower color from orange-red to pale red or even white, with no detectable anthocyanin content in white plants [26]. In pears, *F3H* expression levels are altered as the peel color gradually changes [27]. qRT-PCR analysis of three differently colored mango varieties revealed that *F3H* expression was highest in the red peel of ‘Guifei’, followed by yellow ‘Jinhuang’, and lowest in green ‘Guiqi’ [28].

The late biosynthetic gene *DFR* catalyzes the reduction of colorless dihydroflavonols to colorless leucoanthocyanidins, representing a key rate-limiting step in anthocyanin synthesis, and its expression level directly influences the final anthocyanin yield [26,29,30]. Research indicates a positive correlation between *DFR* expression and the degree of coloration in various fruits, such as apples, pears, and peaches [23,31]. Comparative analysis of *DFR* expression in apple peels with different coloration showed significantly higher *DFR* expression in red-skinned apples than in non-red-skinned apples [32]. ANS is a key enzyme in the later stages of anthocyanin synthesis, belonging to the 2-oxoglutarate-dependent iron dioxygenase family, and catalyzes the conversion of colorless leucoanthocyanidins to colored anthocyanidins [26]. Bagging treatment of the non-red varieties ‘Granny Smith’ and ‘Golden Delicious’ promotes anthocyanin accumulation, which correlates with *UFGT* expression [33]. In Chinese sand pears, *PpUFGT2* expression is closely related to anthocyanin content [34]. In European pears, *UFGT* expression parallels anthocyanin accumulation during fruit development and ripening; however, as fruit approaches full maturity, anthocyanin content declines, while *UFGT* expression does not decrease correspondingly [35].

The accumulation of anthocyanins is not only regulated by genes encoding enzymes involved in their biosynthetic pathway, but is also finely modulated by a network of transcription factors. These regulatory factors mainly include MYB, bHLH, and WD40 repeat proteins, as well as the MYB-bHLH-WD40 (MBW) ternary transcription complexes. By binding to the promoter regions of key genes in the anthocyanin biosynthetic pathway, these transcription factors either activate or repress the expression of these genes, thereby positively or negatively regulating anthocyanin content [36,37]. In apples, the WD40 protein MdTTG1 promotes anthocyanin accumulation. Studies have shown that MdTTG1 interacts with the bHLH transcription factor MdbHLH3 but does not bind to MYB proteins. Given that MdbHLH3 is known to interact with MYB proteins, these findings collectively indicate that MdTTG1 specifically associates with a bHLH protein, rather than with MYB proteins, to regulate anthocyanin accumulation in apples [38]. The R3-type MYB transcription factor MdCPCL interacts with the bHLH transcription factor MdILR3L to form the MdCPCL-MdILR3L complex, which markedly enhances the transcription of downstream target genes, including *MdANS* and *MdUFGT*, thereby promoting anthocyanin synthesis in apples [39]. MdMYB111 and MdWRKY40 form homodimers and bind to the W-box in the *MdANS* promoter, alleviating the inhibition of anthocyanin biosynthesis by MdMYB111, thereby promoting anthocyanin synthesis [40]. The WRKY transcription factor MdWRKY71 interacts with MdMADS1, activating *MdMADS1* transcription and enhancing its transactivation of target genes *MdCHS* and *MdUFGT*. Additionally, MdWRKY71 independently binds and activates the transcription of the anthocyanin biosynthetic genes *MdANS* and *MdDFR*, and the synergistic regulation of anthocyanin biosynthesis by MdWRKY71 and MdMADS1 in apples provides new insights into the molecular control of fruit coloration [41]. Studies have found that the expression level of *UFGT* directly influences anthocyanin content in litchis and strawberries [42].

Apple peel color is a major factor influencing consumer preference and fruit commercial value, with red-skinned cultivars generally commanding higher market acceptance and premium prices [1,2,3]. The ‘Jinhong’ (JH) apple is a hybrid of ‘Golden Delicious’ and ‘Ralls Janet’, and is widely cultivated in northern China for its superior cold hardiness and balanced sweet-sour flavor. However, its relatively light red peel limits its appeal in markets where deep red coloration is preferred. Its natural bud sport strain, ‘Hongjinhong’ (HJH), exhibits a strikingly deep red peel that contrasts sharply with the lighter coloration of JH, thus providing an ideal system for dissecting the molecular mechanisms underlying fruit color variation. Understanding the genetic and metabolic basis of this deeper red coloration is therefore of practical importance for apple breeding programs aimed at enhancing fruit appearance and marketability. However, the key molecular events driving this pronounced color difference remain poorly understood. We hypothesized that the deep red phenotype of HJH apples arises from the specific accumulation of certain anthocyanin species, driven by the upregulation of key structural genes in the anthocyanin biosynthetic pathway. To test this hypothesis, we employed an integrated approach combining physiological and biochemical assays, widely targeted metabolomics, transcriptomics, and qRT-PCR to identify the key metabolites and structural genes associated with the color variation between JH and HJH apples. This study aims to provide novel insights into the regulatory network of apple fruit coloration and to offer candidate genes for the genetic improvement of fruit quality, with potential benefits for consumer-driven apple breeding.

## 2. Results

### 2.1. Deep Red Phenotype of HJH Peel Is Associated with Anthocyanin Accumulation

**Figure 1:**
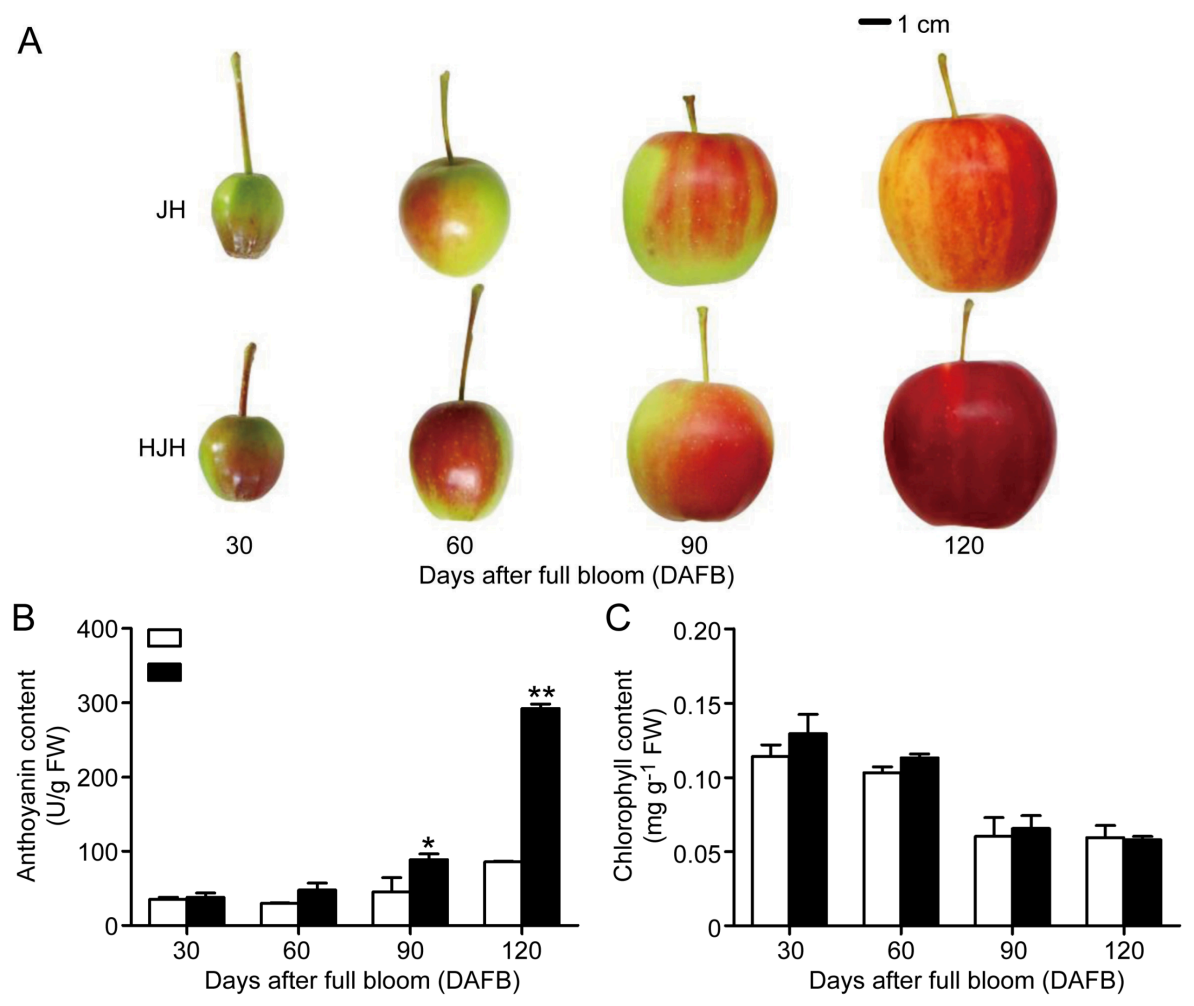
Phenotype and pigment content changes during fruit development in JH and HJH apples. (**A**) Fruit phenotypes at 30, 60, 90, and 120 DAFB, scale bar = 1 cm; (**B**) anthocyanin content; (**C**) total chlorophyll content. Data are means ± SE (*n* = 3), * indicates *p* < 0.05, ** indicates *p* < 0.01 (Student’s *t*-test).

To characterize the color difference between JH and HJH apples, we systematically compared fruit phenotype and peel pigment contents at four developmental stages (30, 60, 90, and 120 days after full bloom (DAFB)). Phenotypic observation revealed that both varieties initiated coloration at 30 DAFB; however, color divergence was already discernible at this early stage. As development progressed, the color contrast became increasingly pronounced. By 120 DAFB, HJH fruits developed a uniform, intense deep red peel, whereas JH fruits exhibited only pale red coloration (Figure 1A). Quantitative analysis of anthocyanin content demonstrated that both varieties underwent an initial decline followed by a progressive increase during fruit maturation. Notably, HJH apples consistently accumulated higher anthocyanin levels than JH apples across all examined time points. The differences were statistically significant at 90 and 120 DAFB (*p* < 0.05), coinciding with the period of maximal anthocyanin accumulation (Figure 1B). In contrast, total chlorophyll content declined steadily throughout fruit development in both genotypes, and no significant differences were detected between JH and HJH apples at any stage (Figure 1C). Collectively, these results indicate that the deep red peel phenotype of HJH apples is primarily attributable to substantially enhanced anthocyanin accumulation, rather than to differential degradation of chlorophyll.

### 2.2. Metabolomic Analysis of Peel Color Differences Between JH and HJH Apples

**Figure 2:**
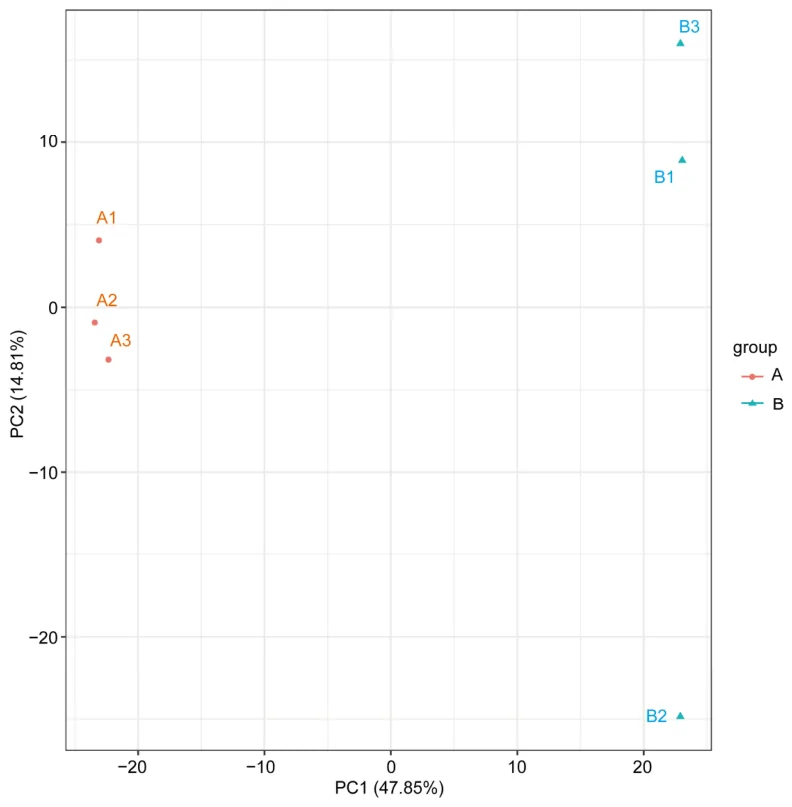
Principal component analysis (PCA) of metabolomic data from JH and HJH peels. JH is designated as A, and HJH as B.

**Figure 3:**
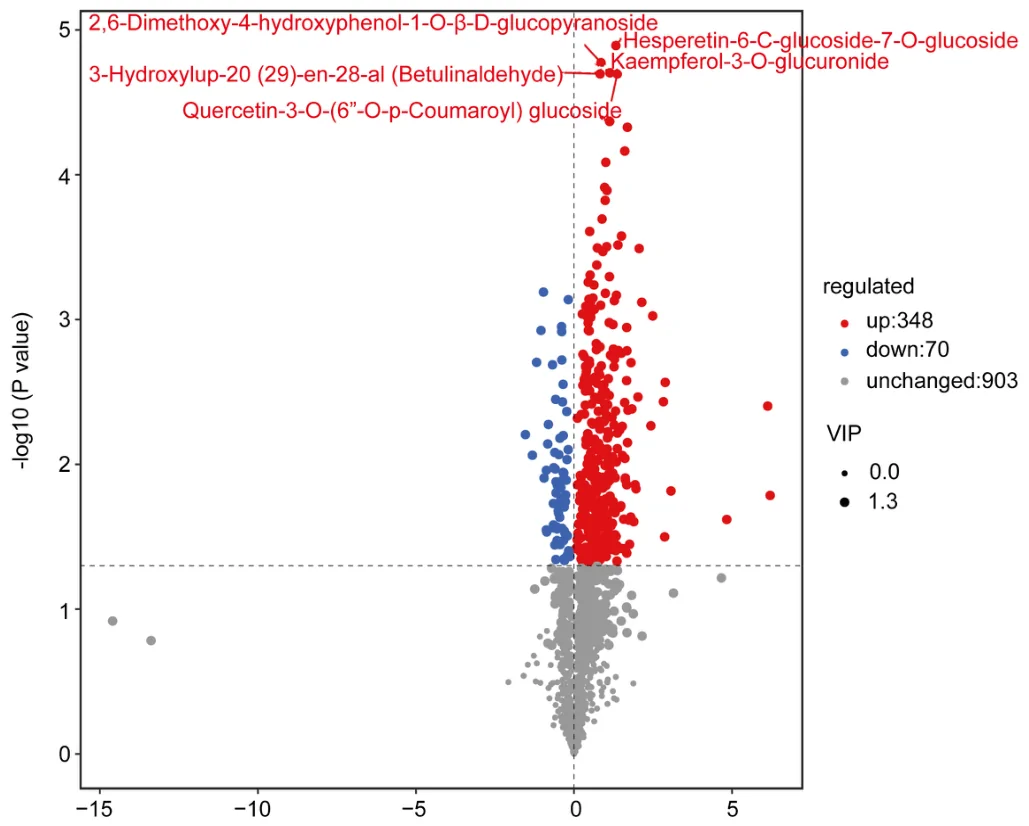
Volcano plot of differentially accumulated metabolites in JH and HJH peels (with JH as the control). Red dots represent metabolites significantly upregulated in HJH peels (log_2_FC ≥ 1, VIP ≥ 1), green dots represent metabolites significantly downregulated in HJH peels (log_2_FC ≤ −1, VIP ≥ 1), and gray dots represent metabolites with no significant difference. Thresholds: VIP ≥ 1 and |log_2_FC| ≥ 1.

To investigate the metabolites contributing to the peel color differences between JH and HJH apples, widely targeted metabolomic analysis was performed on peel samples collected at 90 DAFB, with three biological replicates per sample. Principal component analysis (PCA) was employed to evaluate the overall metabolic variation between the two cultivars. The PCA score plot showed that the first principal component (PC1) explained 63.1% of the total variance, while the second principal component (PC2) accounted for 9.67%, together explaining 72.77% of the cumulative variance. The samples from JH and HJH apples were clearly separated along PC1 and PC2, indicating substantial differences in their metabolic profiles (Figure 2). Differentially accumulated metabolites (DAMs) were identified using thresholds of Variable Importance in Projection (VIP) ≥ 1 and |Fold Change| ≥ 2. A total of 418 DAMs were screened, comprising 348 upregulated and 70 downregulated metabolites in HJH relative to JH apples, as depicted in the volcano plot (Figure 3).

**Figure 4:**
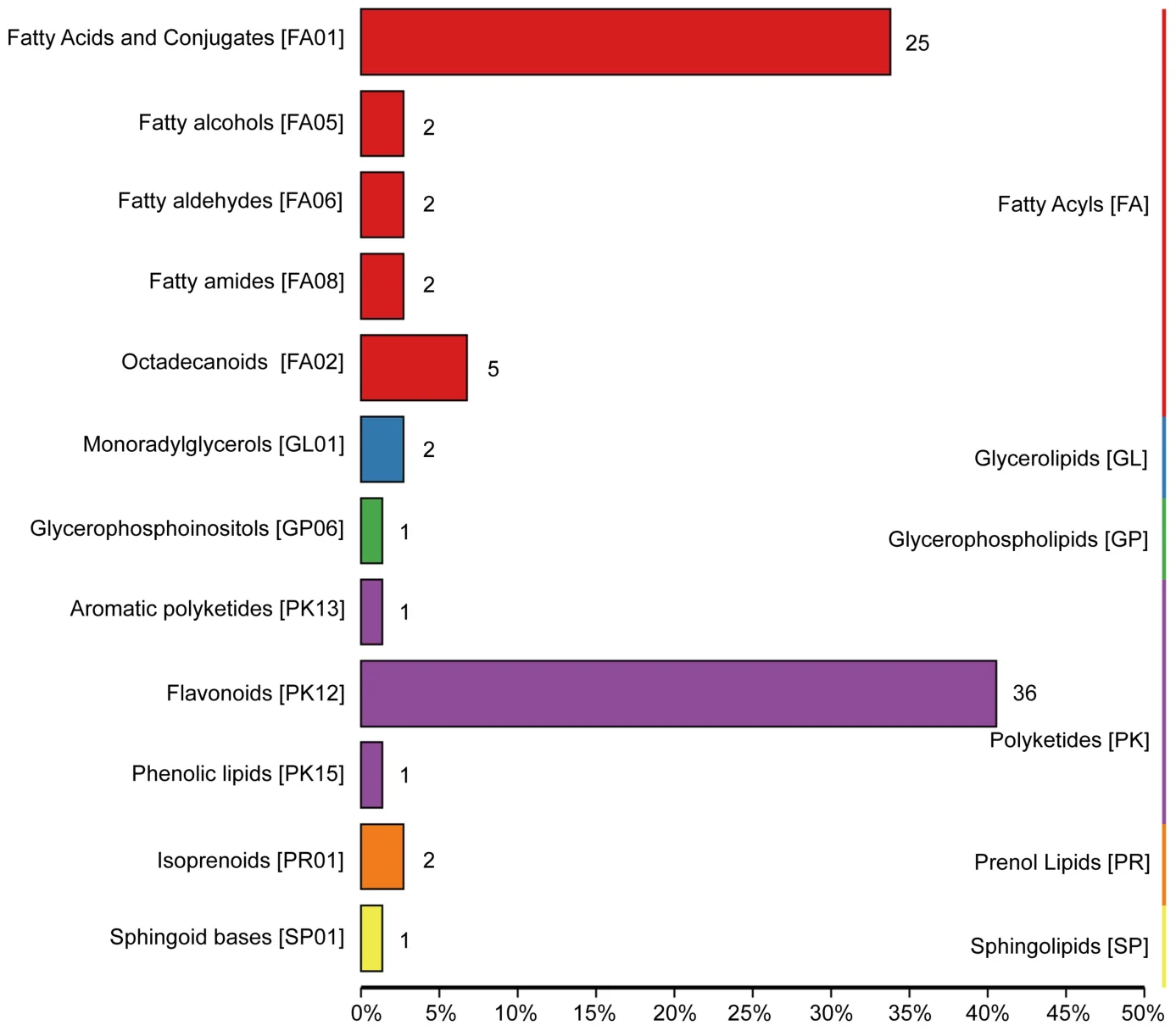
LIPID MAPS hierarchical classification of metabolites in JH and HJH peels. Entries in the same box represent LIPID MAPS hierarchical classification information, corresponding to the CATEGORY and MAIN CLASS information in the LIPID MAPS database. Bar length indicates the number of metabolites annotated to that category.

LIPID MAPS (Lipid Metabolites and Pathways Strategy) is a database that classifies biological lipids into eight major categories. LIPID MAPS analysis showed that the identified metabolites were mainly concentrated in fatty acyls, with fatty acids and conjugates being the most abundant, and in polyketides, with flavonoids being the most abundant (Figure 4).

**Figure 5:**
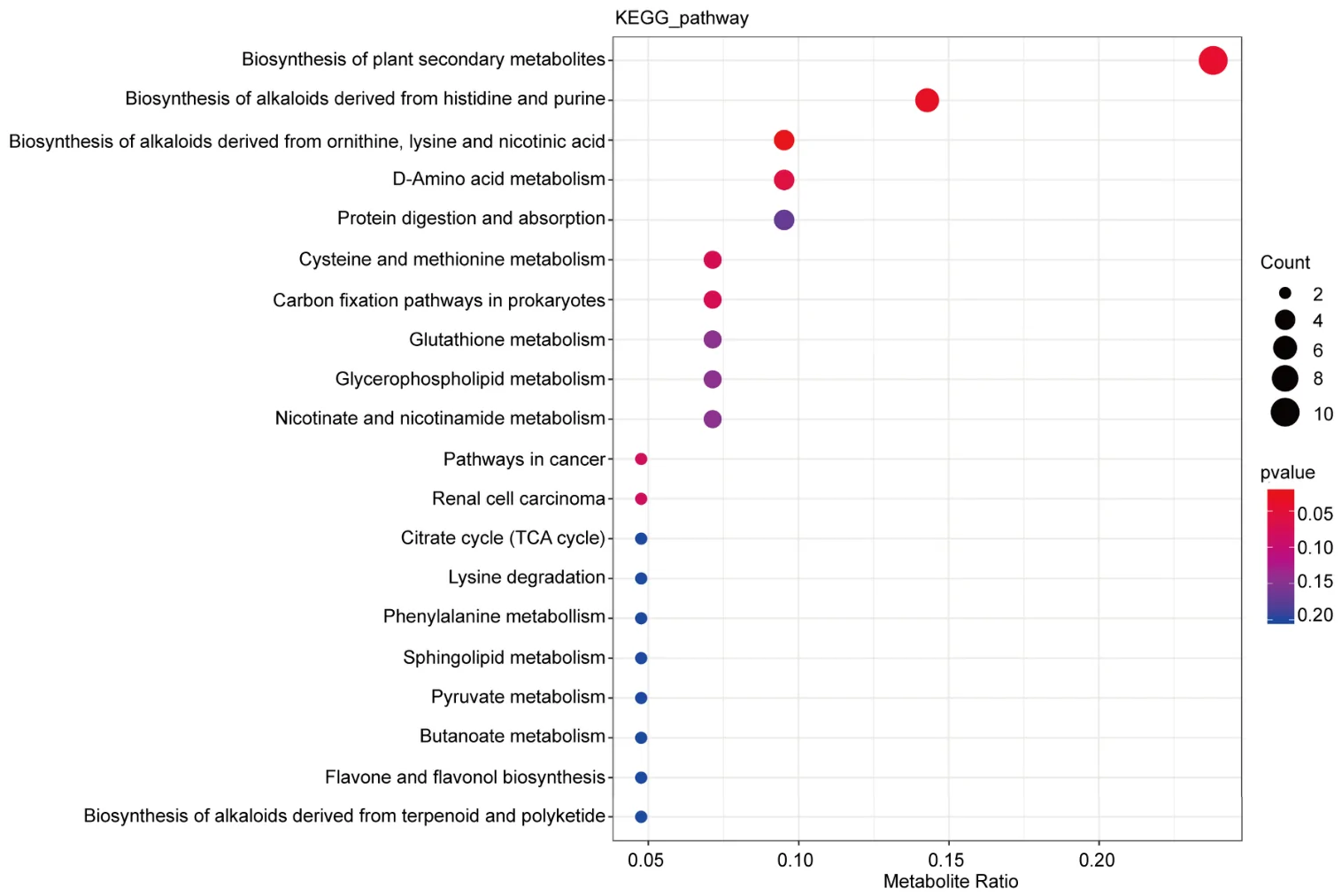
KEGG pathway enrichment analysis of differentially accumulated metabolites in JH and HJH peels.

To further investigate the functions of differential metabolites, the top 20 enriched KEGG (Kyoto Encyclopedia of Genes and Genomes) pathways were analyzed. Differential metabolites were enriched in several metabolic pathways, including biosynthesis of secondary metabolites, biosynthesis of histidine and purine-derived alkaloids, biosynthesis of ornithine, lysine, and nicotinic acid alkaloids, and flavonoid and flavonol biosynthesis (Figure 5).

### 2.3. Analysis of Major Metabolites Reveals Cyanidin Derivatives as Key Differential Metabolites Between JH and HJH Apples

A total of 362 flavonoid metabolites were detected in peel samples from JH and HJH apples during fruit development (Table 1). Among these, flavonols and flavones were the most abundant classes, with 137 and 87 metabolites, respectively, while isoflavones were the least abundant, with only 3 metabolites detected. Nine anthocyanins were detected. Other detected metabolite classes included 31 dihydroflavones, 28 flavanols, 22 chalcones, 15 tannins, 13 proanthocyanidins, 10 dihydroflavonols, and 7 other flavonoids, comprising 11 classes in total.

KEGG pathway enrichment analysis of the DAMs (Table 2) revealed significant enrichment in several pathways, including biosynthesis of secondary metabolites, flavonoid and flavonol biosynthesis, flavonoid biosynthesis, and anthocyanin biosynthesis. Within the anthocyanin biosynthesis pathway, three cyanidin-type anthocyanins were identified as DAMs, all of which were significantly more abundant in HJH than in JH apples: cyanidin-3-O-(6″-O-malonyl) glucoside (Log_2_FC = 2.83, FC = 7.11, VIP = 1.41), cyanidin-3-O-(2″-O-xylosyl) galactoside (Log_2_FC = 1.71, FC = 3.26, VIP = 1.43), and cyanidin-3-O-glucoside (Log_2_FC = 1.31, FC = 2.49, VIP = 1.41). Among these, cyanidin-3-O-(6″-O-malonyl) glucoside exhibited the most pronounced fold change, indicating that it is the primary pigment responsible for the deeper red coloration of HJH peel. In the flavonoid biosynthesis pathway, four metabolites were identified as DAMs (leucocyanidin, myricetin, gallocatechin, and epigallocatechin), all of which were upregulated in HJH. These results collectively suggest that the substantial accumulation of cyanidin-type anthocyanins, particularly cyanidin-3-O-(6″-O-malonyl) glucoside, constitutes the key metabolic basis for the color difference between the two cultivars.

### 2.4. Transcriptome Volcano Plot Analysis

Based on our previous findings that peel color deepened progressively during fruit development and that anthocyanin content began to accumulate substantially at 90 DAFB, we performed transcriptome sequencing analysis on peel samples from JH and HJH apples collected at 90 DAFB, with three replicates per sample. The results showed that a total of 36.17 Gb of clean data were obtained from JH and HJH peel samples, with GC content exceeding 46.89% and the percentage of Q30 bases not less than 92.51% for each sample (Table 3).

**Figure 6:**
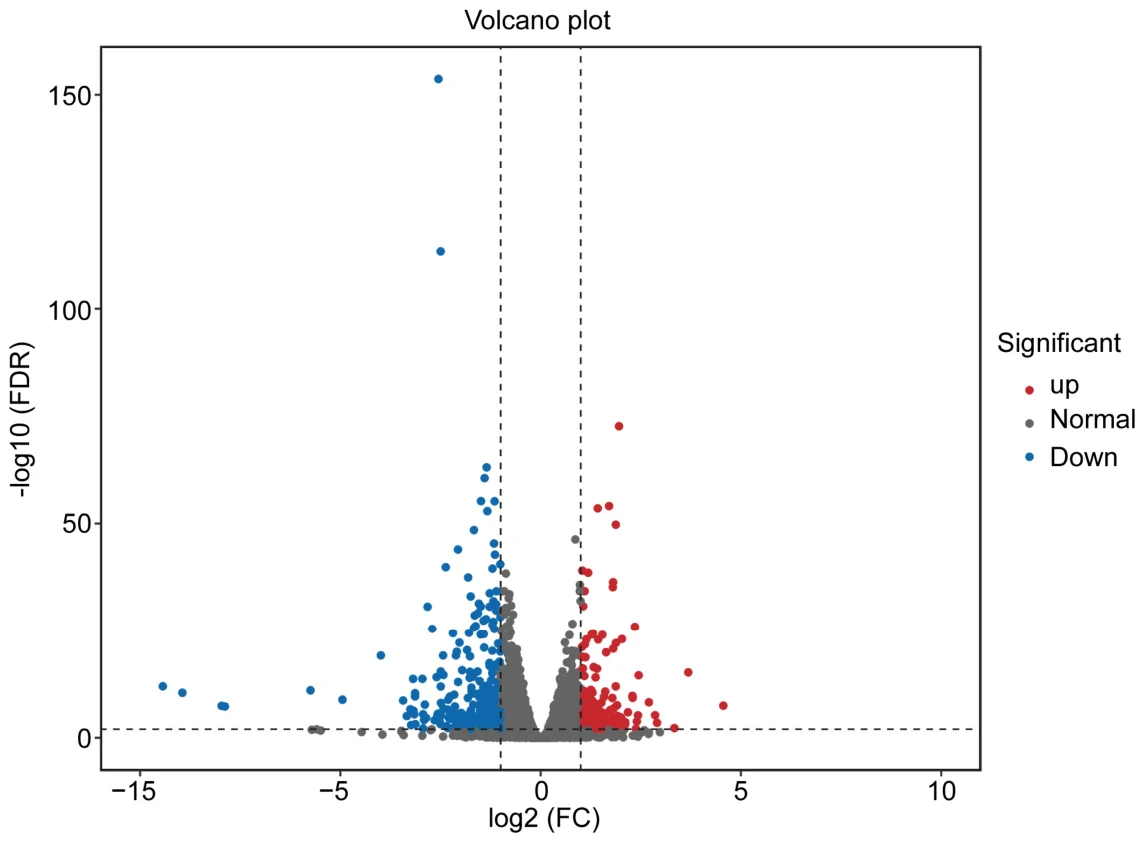
Volcano plot of transcriptome data from JH and HJH peels (with JH as the control). Red dots represent genes significantly upregulated in HJH peels (log_2_FC ≥ 1, FDR < 0.05), green dots represent genes significantly downregulated in HJH peels (log_2_FC ≤ −1, FDR < 0.05), and gray dots represent genes with no significant difference. Thresholds: |log_2_FC| ≥ 1 and FDR < 0.05.

Volcano plots effectively reflect both the magnitude of gene expression differences and their statistical significance between two samples. To identify genes regulating the differential accumulation of anthocyanins between JH and HJH apples, we performed volcano plot analysis of the peel transcriptome data. Using thresholds of |log_2_FC| ≥ 1 and FDR < 0.05 for preliminary screening of DEGs (differentially expressed genes), a total of 533 DEGs were identified, comprising 209 upregulated genes and 324 downregulated genes (Figure 6).

### 2.5. GO Enrichment Analysis of Transcriptome Data

**Figure 7:**
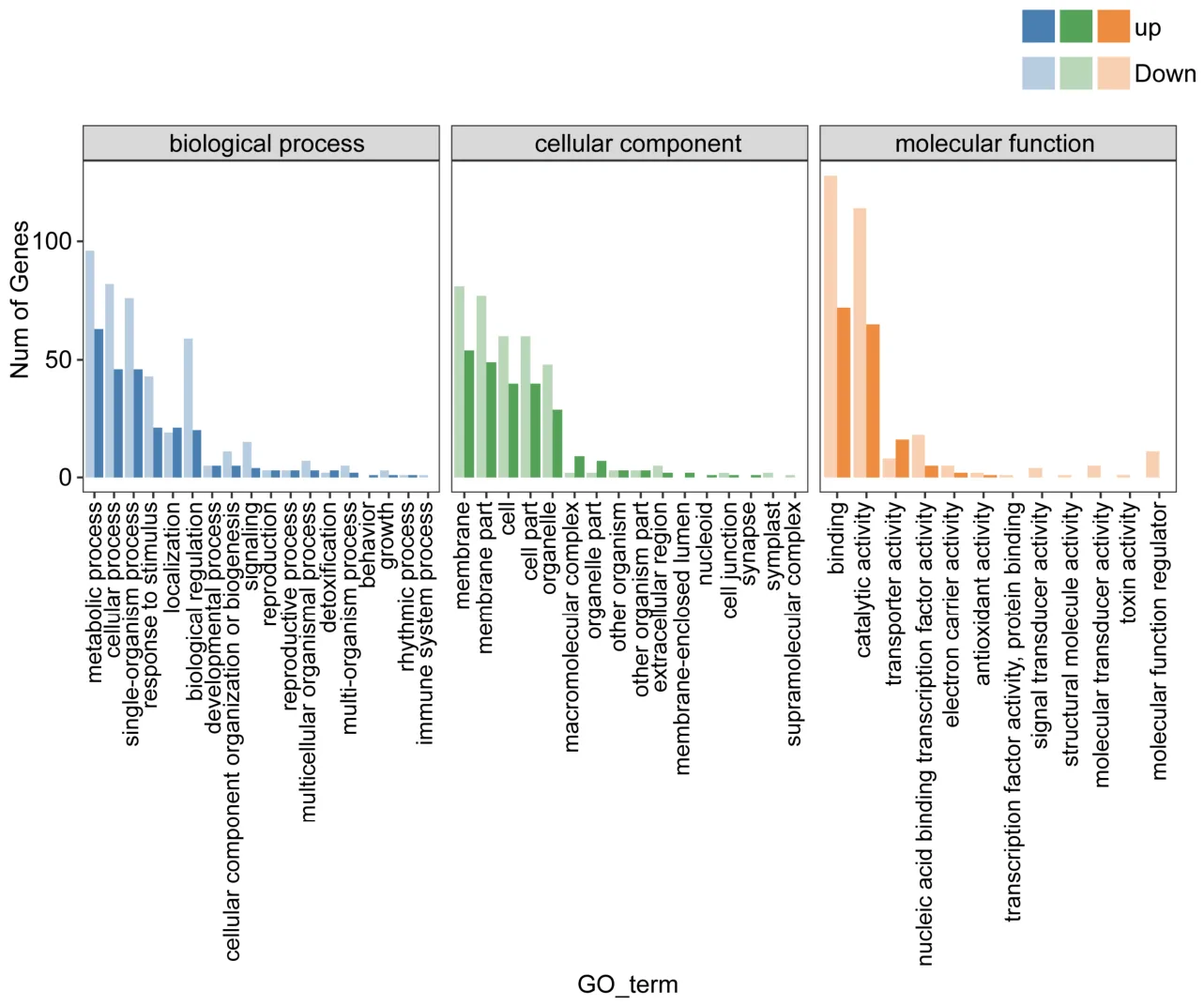
GO enrichment analysis of transcriptome data from JH and HJH peels. The x-axis presents the category names, the y-axis presents the numbers of genes, and different colors indicate different primary categories.

To functionally categorize the differentially expressed genes (DEGs) between JH and HJH peels, Gene Ontology (GO) enrichment analysis was performed. A total of 426 DEGs were assigned to 46 functional groups, which were further classified into three main ontologies: biological process (BP), cellular component (CC), and molecular function (MF) (Figure 7). Within the BP category, the most enriched subcategories were “metabolic process” and “cellular process”. The enrichment of metabolic process-related genes is biologically meaningful, as it reflects the enhanced secondary metabolic activities, particularly the flavonoid and anthocyanin biosynthetic pathways, which are central to the accumulation of red pigments in HJH peel. The dominance of “catalytic activity” and “binding” in the MF category further supports this interpretation, as these terms include enzymes (e.g., transferases, oxidoreductases) and regulatory proteins that directly participate in anthocyanin biosynthesis and its transcriptional regulation. In the CC category, the enrichment of genes associated with “membrane” and “membrane part” suggests that subcellular compartmentalization and transport processes may also play roles in the sequestration and stabilization of anthocyanins, which are typically stored in vacuoles following transport across endomembranes. Collectively, these GO enrichment patterns indicate that the observed transcriptional reprogramming in HJH peel is functionally linked to enhanced metabolic flux through the anthocyanin biosynthetic pathway, thereby providing a molecular basis for its deeper red coloration.

### 2.6. KEGG Enrichment Analysis of Transcriptome Data

**Figure 8:**
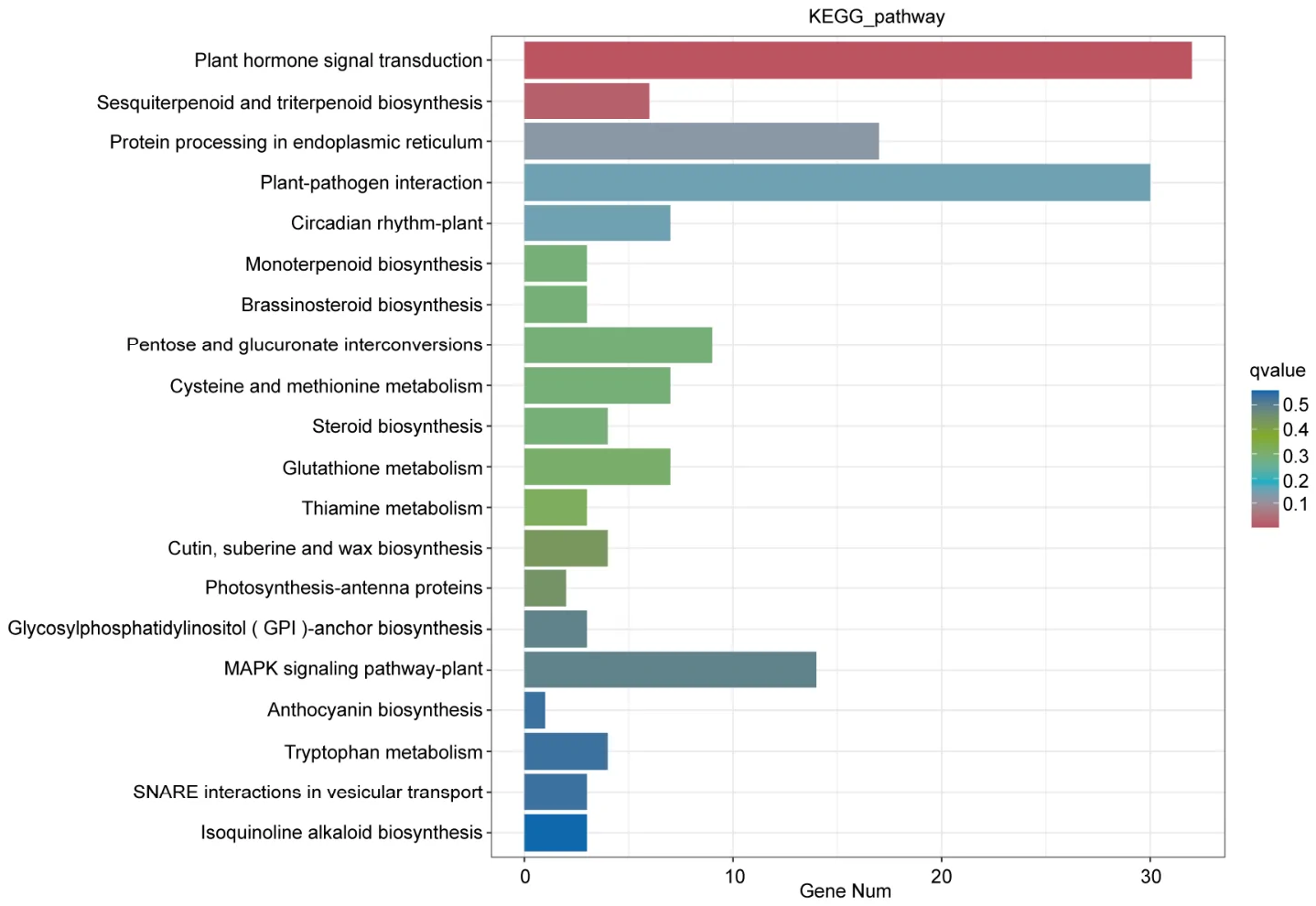
KEGG enrichment analysis of transcriptome data from JH and HJH peels. The x-axis presents the numbers of genes in the pathway, and the y-axis presents the pathway annotations. Bar color indicates the q-value from the hypergeometric test.

KEGG is widely used for analyzing gene functions, mining genomic information, and interpreting gene functions. Analysis of the top 20 enriched KEGG pathways revealed that DEGs were enriched in pathways such as plant–pathogen interaction, plant hormone signal transduction, and anthocyanin biosynthesis (Figure 8).

Further screening of DEGs within the anthocyanin biosynthesis pathway from the KEGG enrichment analysis identified five differential structural genes associated with anthocyanin biosynthesis, including *MdDFR*, *MdCHS*, *MdUFGT*, *MdF3’H* (flavonoid 3’-hydroxylase), and *MdLAR* (leucoanthocyanidin reductase). Therefore, these genes were considered potential contributors to the fruit color difference between JH and HJH apples (Table 4).

### 2.7. Integrated Transcriptomic and Metabolomic Analysis

**Figure 9:**
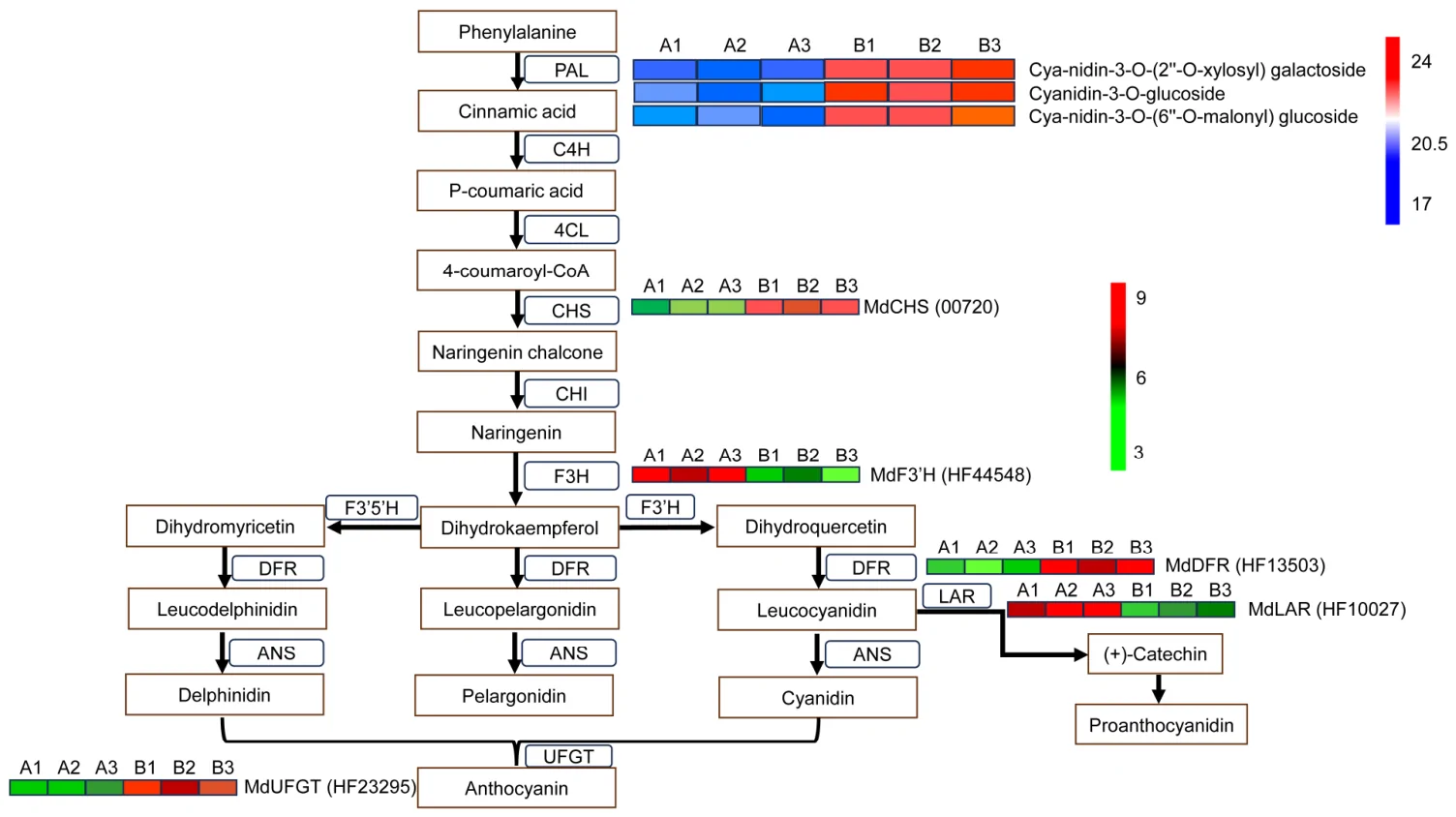
Correlation analysis between DEGs and DAMs in the anthocyanin biosynthesis pathway. JH is designated as A, and HJH as B.

To explore the correlation between the transcriptome and metabolome in the peels of JH and HJH apples, a correlation analysis was performed using DEGs and DAMs during the process of peel color change. Several structural genes and metabolites were significantly enriched in the anthocyanin biosynthesis pathway, suggesting that metabolite accumulation is specifically regulated by DEGs. Among the identified metabolites, three anthocyanins were present at higher levels in the HJH variety, namely cyanidin-3-O-glucoside, cyanidin-3-O-(2″-O-xylosyl) galactoside, and cyanidin-3-O-(6″-O-malonyl) glucoside. Regarding gene expression, *MdDFR*, *MdCHS*, *MdUFGT*, *MdF3’H*, and *MdLAR* exhibited distinct correlation patterns with different metabolites, including both positive and negative correlations. Specifically, *MdDFR*, *MdCHS*, and *MdUFGT* were predominantly positively correlated with the metabolites, whereas *MdF3’H* and *MdLAR* showed negative correlations (Figure 9).

### 2.8. Validation of DEGs by qRT-PCR

**Figure 10:**
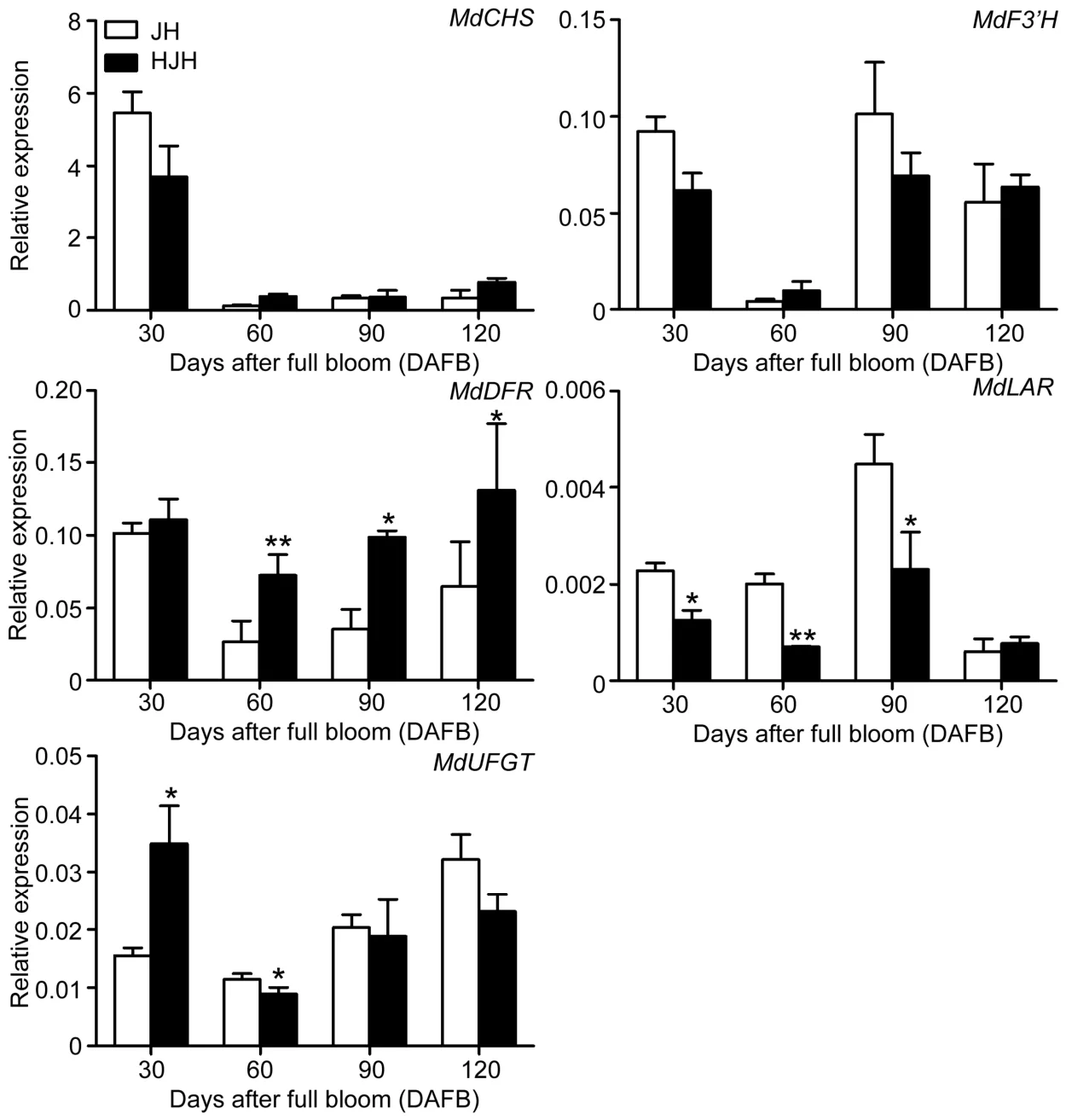
Expression analysis of anthocyanin biosynthetic structural genes during fruit development of JH and HJH apples. Data are presented as mean ± standard error (*n* = 3), * *p* < 0.05, ** *p* < 0.01 (Student’s *t*-test).

To further investigate the expression dynamics of the five anthocyanin biosynthetic structural genes identified by transcriptomic analysis, qRT-PCR was performed on peel samples collected at four developmental stages (30, 60, 90, and 120 DAFB) from both JH and HJH fruits (Figure 10). The results revealed distinct and gene-specific expression patterns. *MdCHS* and *MdF3’H* showed no statistically significant differences between HJH and JH apples at any of the four time points, suggesting that their transcriptional activation is not a primary determinant of the enhanced red coloration in HJH apples. *MdUFGT* expression was significantly higher in HJH than in JH apples only at 30 DAFB, and higher in JH apples at 60 DAFB, with no differences at later stages, indicating that its expression does not consistently correlate with the final anthocyanin accumulation pattern. *MdLAR* expression was significantly higher in JH than in HJH apples at 30, 60, and 90 DAFB, and only at 120 DAFB did the difference disappear. Given that *LAR* encodes leucoanthocyanidin reductase, which directs the flavonoid flux toward proanthocyanidin synthesis, its higher expression in JH apples may divert precursors away from the anthocyanin branch. In contrast, *MdDFR* exhibited a consistent and biologically relevant expression pattern. No significant difference was observed at 30 DAFB, when anthocyanin levels were still low in both varieties. However, from 60 DAFB onward, *MdDFR* expression was significantly higher in HJH than in JH apples. Moreover, the expression trend of *MdDFR* in both varieties––declining from 30 to 60 DAFB and then progressively increasing toward ripening––closely mirrored the temporal trajectory of the total anthocyanin content measured in the peels (Figure 1B). This strong positive correlation between *MdDFR* transcript abundance and anthocyanin accumulation, together with the metabolomic finding that upstream leucoanthocyanidin levels were elevated in HJH apples, identifies *MdDFR* as the most likely rate-limiting structural gene responsible for the enhanced synthesis of cyanidin-type anthocyanins in HJH peel.

## 3. Discussion

Apple color is a key quality trait determining commercial value [1,2,3], with red-fleshed or red-skinned varieties being particularly favored by consumers due to their high anthocyanin content [20,43]. Although the mechanisms underlying anthocyanin biosynthesis and regulation have been extensively studied in various plant species [20], the molecular basis of fruit color differences between varieties or germplasms remains largely unknown. Apple peel coloration is determined by the combined effects of three classes of pigments––anthocyanins, chlorophylls, and carotenoids––with the final hue depending on the relative proportions and spatial distribution of these components [44]. Studies have shown that, even at comparable anthocyanin concentrations, residual chlorophyll levels significantly influence the resulting coloration: higher chlorophyll content results in a darker red hue, whereas lower chlorophyll content allows for a brighter, more vivid red [43,45,46,47]. In ‘Royal Gala’ apples, for example, the development of red color during ripening is attributed not only to anthocyanin biosynthesis, but also to substantial chlorophyll degradation [48]. Carotenoids, as important terpenoid compounds, are fundamental pigments responsible for yellow peel color, directly influencing fruit background color. In the ‘OPAL^®^’ variety, IAA-mediated degreening during ripening promotes gradual chlorophyll degradation, allowing carotenoids to become visible and the peel to transition to a yellow background color [48]. Due to variations in pigment accumulation characteristics and metabolic regulatory mechanisms among different varieties, apple peels display a diverse spectrum of colors ranging from verdant green and golden yellow to deep purple. This multi-pigment coordinated regulatory mechanism explains the diversity of apple peel colors. In this study, analysis of chlorophyll content in JH and HJH apples revealed no differences between the two, whereas anthocyanin content differences were responsible for the fruit color variation (Figure 1). This finding aligns with previous reports indicating that significant differences in the specific types and relative abundances of anthocyanins among apple varieties contribute to the diverse range of hues observed, from light pink and bright red to deep purple [49].

Color is a key determinant of apple quality, and the mechanisms underlying its formation have been a focal point of pomology research [20]. To date, over 600 anthocyanins have been identified in nature [13]. The most common natural anthocyanins include cyanidin, delphinidin, malvidin, petunidin, peonidin, and pelargonidin, among which cyanidin and its derivatives are the most prevalent [13,14]. In apple peels, five major anthocyanin components are present: cyanidin-3-xyloside, cyanidin-3-rutinoside, cyanidin-3-arabinoside, cyanidin-3-galactoside, and cyanidin-3-glucoside [16,17]. This study found that the anthocyanin content in HJH peel was substantially higher than in JH peel (Figure 1), and this difference was primarily attributable to the massive accumulation of cyanidin derivatives, particularly cyanidin-3-O-(6″-O-malonyl) glucoside (Table 2). This finding is consistent with previous reports that cyanidin is the predominant anthocyanin type in the peel of Rosaceae fruit trees such as apple and pear [16,17,50,51]. Some studies suggest that cyanidin-3-galactoside is the most abundant anthocyanin in apples, accounting for over 85% of total anthocyanin content, with other pigments including cyanidin-3-arabinoside and cyanidin-3-glucoside, among others [50]. In this study, three differential metabolites in the anthocyanin biosynthesis pathway––cyanidin-3-O-glucoside, cyanidin-3-O-(2″-O-xylosyl) galactoside, and cyanidin-3-O-(6″-O-malonyl) glucoside––were all more abundant in HJH apples, with cyanidin-3-O-(6″-O-malonyl) glucoside showing massive accumulation (Table 2). These results indicate that both the type and content of anthocyanins determine fruit color.

In the present metabolomic analysis, the malonylated anthocyanin cyanidin-3-O-(6″-O-malonyl) glucoside exhibited the highest fold change (FC = 7.11) among all identified differentially accumulated metabolites (Table 2). This result not only reveals the material basis underlying the deeper red coloration of the HJH peel, but also highlights the critical role of malonylation modification in apple peel color formation. Malonylation is an important acylation modification of anthocyanins, catalyzed by malonyltransferase (MaT). This enzyme uses malonyl-CoA as a donor and specifically acts on the hydroxyl group at the 6″-position of anthocyanidin-3-O-β-D-glucoside to convert it into a 6″-O-malonylated derivative. Extensive research has demonstrated that malonylation plays important regulatory roles in anthocyanin stability and biological function. Regarding chemical stability, cyanidin 3-O-6″-O-malonylglucoside recombinantly produced by a dahlia cDNA-encoded malonyltransferase exhibited significantly higher spectral stability at physiological pH than its non-malonylated precursor cyanidin 3-O-glucoside, and 6″-O-malonylation effectively protected the anthocyanin from degradation by β-glucosidase [52]. A comparative analysis of various cyanidin C-ring modifications further revealed that malonylation conferred superior anthocyanin retention and color stability compared to glucosylation alone in an apple juice beverage matrix [53]. Moreover, beyond its stabilizing effect, malonylation also promotes the vacuolar transport and sequestration of anthocyanins. In *Medicago truncatula*, malonylation was shown to significantly increase both the affinity and the transport efficiency of flavonoid glucosides for uptake by the MATE2 vacuolar transporter [54]. In apples, the transcription factor MdMYB1 directly regulates the expression of a series of transporters, including MATE family members, thereby facilitating the vacuolar sequestration of anthocyanins and malate [55]. Collectively, these findings suggest that the substantial accumulation of cyanidin-3-O-(6″-O-malonyl) glucoside in the HJH peel likely exerts dual biological effects. On the one hand, the 6″-O-malonyl modification introduces a malonyl group to the C-ring sugar moiety, enhancing the overall chemical stability of the molecule and rendering it less susceptible to degradation, thereby maintaining the vibrant red color of the peel over extended periods. On the other hand, malonylation significantly improves the affinity of anthocyanin glycosides for vacuolar MATE transporters and their transport efficiency, promoting efficient and targeted sequestration into the vacuole, thus enabling effective intracellular accumulation and stable storage of anthocyanins. In the future, the identification of specific anthocyanin malonyltransferases in apples and the elucidation of the regulatory mechanisms governing their encoding genes will represent important research directions for a deeper understanding of red color formation and maintenance in apple peel.

Anthocyanin biosynthesis is a crucial branch of the flavonoid metabolic pathway, starting from the substrate phenylalanine and proceeding through a series of enzymatic reactions to ultimately form stable anthocyanins. Key enzymes involved in this pathway include PAL, C4H (cinnamate-4-hydroxylase), 4CL (4-coumaroyl-CoA ligase), CHS, CHI, F3H, F3’H, F3’5’H (flavonoid 3′,5′-hydroxylase), DFR, ANS, and UFGT [29,56,57,58]. In this study, we screened for differentially expressed genes within the anthocyanin biosynthetic pathway based on KEGG enrichment analysis of the transcriptome and identified five differentially expressed structural genes involved in anthocyanin biosynthesis: *MdDFR*, *MdCHS*, *MdUFGT*, *MdF3’H*, and *MdLAR* (Table 4). The expression characteristics and functions of structural genes in the anthocyanin biosynthesis pathway have been extensively studied in apples. As the rate-limiting enzyme catalyzing the initial step of the pathway, PAL activity in apple fruit exhibits two peaks during development, occurring in the young fruit stage and the mature stage; however, PAL activity correlates positively with anthocyanin accumulation only during the young fruit stage, with no apparent association during the mature stage [59]. CHS is responsible for constructing the basic carbon skeleton of flavonoids and is a key enzyme in flavonoid metabolism. In apples, the expression levels of *CHS* and other structural genes in red varieties such as ‘Jonagold’ and ‘Fuji’ are significantly higher than in the non-red variety ‘Orin’ [31]; however, *CHS* maintains relatively high expression throughout fruit development (from young fruit to maturity), and light treatment does not affect anthocyanin synthesis by regulating *CHS* [60]. During the fruit coloring stage, changes in anthocyanin content in the peel of the red apple variety ‘Splendour’ correlate positively with *CHI* expression levels [61]. In this study, qRT-PCR analysis of four developmental stages of JH and HJH fruits revealed that, although *CHS* showed differences starting at 60 DAFB, these differences were not statistically significant (Figure 10).

F3H catalyzes the conversion of naringenin to dihydrokaempferol (DHK), a reaction involved not only in anthocyanin synthesis, but also in providing precursors for downstream pathways. During the coloring stage of red apple varieties, *F3H* transcript levels are significantly higher than in non-red varieties, suggesting a key role for F3H in apple peel color development [62]. Dihydrokaempferol is subsequently converted to dihydroquercetin (DHQ) and dihydromyricetin (DHM) by F3’H and F3’5’H, respectively [29,30]. In the presence of the coenzyme NADPH, DFR catalyzes the reduction of dihydroflavonols (DHK, DHQ, DHM) to their corresponding leucoanthocyanidins [26]. Studies have shown that *DFR* expression levels are significantly higher in red apple varieties than in non-red varieties [62]. DFR is the first rate-limiting enzyme that specifically channels the pathway toward colored anthocyanins, and its expression level and substrate preference directly influence the yield and type of downstream anthocyanins [29,30]. In this study, *MdDFR* showed no significant difference at 30 DAFB but was highly expressed in HJH apples at all other stages. The expression trend of *MdDFR* in both JH and HJH apples initially decreased and then increased, consistent with the pattern of anthocyanin content changes in the peels of both varieties. Moreover, in the metabolomic data, the content of cyanidin pigments was influenced by the level of upstream leucoanthocyanidins. The sustained high expression of *MdDFR* in HJH apples likely provides an adequate supply of precursors (leucoanthocyanidins) for the substantial synthesis of cyanidin-type pigments (Figure 10). This finding is consistent with studies in red-skinned pears and red-fleshed apples, which also indicate that upregulation of *DFR* is a key molecular event underlying red color formation [63,64]. Therefore, identifying *MdDFR* as a regulatory target is crucial for dissecting the upstream regulatory mechanisms. Subsequent research will focus on identifying transcription factors involved in *MdDFR* regulation and verifying their interactions with this structural gene via biochemical assays, thus revealing the transcriptional network responsible for the elevated *MdDFR* expression in HJH. Nevertheless, it is important to acknowledge that the conclusion regarding *MdDFR* as the core molecular event is currently based on correlative evidence (expression trends matching anthocyanin accumulation) rather than direct functional validation. Correlation does not imply causation. Future studies employing transient transformation, stable genetic manipulation, or gene editing are necessary to establish the causal role of *MdDFR* in the deep red phenotype. Despite this limitation, the present study provides a robust correlative framework and identifies promising candidate genes for further mechanistic exploration.

The apple genome contains genes such as *LAR1* and *LAR2*, which encode LAR enzymes responsible for catalyzing the conversion of leucoanthocyanidins to catechins, a key step in proanthocyanidin (condensed tannin) synthesis [65]. In addition, MdbHLH33 binds to the low-temperature responsive element (LTR) in the promoter of *MdMYBPA1*, thereby promoting the conversion of proanthocyanidins to anthocyanins [66]. In contrast to the anthocyanin accumulation trend, *MdLAR* transcript levels were significantly higher in JH than in HJH apples at most developmental stages (Figure 10). Given that *LAR* catalyzes the conversion of leucoanthocyanidins to proanthocyanidins, the elevated *MdLAR* expression in JH apples may divert the flavonoid flux away from anthocyanin biosynthesis, thereby partially accounting for the lighter red phenotype of JH apples. UFGT catalyzes the final step of the anthocyanin synthesis pathway, stabilizing unstable anthocyanidins into anthocyanins through glycosylation. During different stages of apple development, although the expression pattern of *UFGT* may vary, its transcript levels consistently correlate with the trend of anthocyanin accumulation [67]. In this study, *MdUFGT* was highly expressed in HJH apples only at 30 DAFB and in JH apples at 60 DAFB, with no significant differences at other stages (Figure 10).

## 4. Materials and Methods

### 4.1. Plant Materials and Treatments

JH and its red bud sport strain HJH apple trees grafted on *Malus baccata* rootstock were cultivated in the orchard of Mudanjiang Branch, Heilongjiang Academy of Agricultural Sciences, Mudanjiang City, Heilongjiang Province (129°30′18″ E, 44°24′59″ N). Fruits of uniform size and free from pests and diseases were collected at 30, 60, 90, and 120 DAFB. Fruits were transported to the laboratory on the same day. The peels were carefully excised, immediately immersed in liquid nitrogen for rapid freezing, then collected using a slotted spoon, placed into sealed plastic bags, and stored at −80 °C for subsequent analysis.

### 4.2. Determination of Anthocyanin and Chlorophyll Contents

Anthocyanin content was measured according to a previously described method, with minor modifications [68]. Peel samples (0.3 g) were extracted with 1 mL of methanol containing 1% HCl overnight at room temperature in the dark. After centrifugation, the supernatant was collected, and absorbance was measured at 553 nm and 600 nm using a UV-2600 spectrophotometer (Shimadzu, Kyoto, Japan). Relative anthocyanin content was expressed as (A553-A600)/0.01·g^−1^·FW (U). Chlorophyll content was determined following the method of Salah et al. [69]. Peel samples (0.2 g) were extracted with 10 mL of a mixture of 95% ethanol and 80% methanol (1:1, *v*/*v*) for 24 h in the dark at room temperature. Absorbance was measured at 665 nm, 649 nm, and 470 nm to calculate chlorophyll a, chlorophyll b, and total chlorophyll contents. Three biological replicates were used for each assay.

### 4.3. Metabolome and Transcriptome Sequencing Analysis

Peel samples from JH and HJH at 90 DAFB (a stage at which the peel color difference between the two cultivars is significant), each with three biological replicates, were submitted to Biomarker Technologies Co., Ltd. (Beijing, China) for metabolomic and transcriptomic sequencing. Metabolite profiling was performed using an LC-ESI-MS/MS system, and data were analyzed based on an in-house database and public databases. Differential metabolites were selected based on VIP ≥ 1 and a fold change ≥ 2 or ≤ 0.5. Transcriptome sequencing was conducted on the Illumina platform. Raw reads were filtered and mapped to the apple reference genome (GDDH13 Version 1.1) (https://iris.angers.inra.fr/gddh13/) (9 November 2022). Differentially expressed genes (DEGs) were identified with thresholds of FDR < 0.05 and |log_2_FC| ≥ 1.

### 4.4. RNA Extraction, cDNA Synthesis, and Quantitative Real-Time PCR (qRT-PCR)

Total RNA was extracted from peel samples using a modified CTAB method. RNA concentration and integrity were assessed using a Nanodrop 2000 (Thermo, Waltham, MA, USA) and agarose gel electrophoresis. cDNA was synthesized using the PrimeScript™ RT Reagent Kit with gDNA Eraser (RR047A, Takara, Dalian, China) following the manufacturer’s instructions, as previously described [70]. Primers were designed using Primer 3.0 (https://primer3.ut.ee/) (25 November 2022), and the primer sequences are listed in Supplementary Table S1. The apple *Actin* gene was used as an internal control, and relative gene expression was calculated using the 2^−ΔΔCt^ method. Three biological replicates were used per sample. qRT-PCR was performed using UltraSYBR Mixture (CW0957S, CWBIO, Taizhou, China) according to the manufacturer’s instructions. The total reaction volume was 10 μL, containing 5 μL of 2× UltraSYBR Mixture (Low ROX), 0.5 μL of cDNA, 0.5 μL each of forward and reverse primers, and 3.5 μL of water. The reaction was carried out on a LightCycler^®^ 96 system (Roche, Basel, Switzerland) under the following conditions: 95 °C for 3 min, followed by 33 cycles of 95 °C for 30 s, 55 °C for 30 s, and 72 °C for 30 s, with a final dissociation stage at 72 °C for 30 s.

### 4.5. Statistical Analysis

All physiological and molecular data were obtained from at least three independent biological replicates, each with three technical replicates. Results are presented as mean ± standard error of the mean (SEM). Prior to statistical comparisons, data normality was assessed using the Shapiro-Wilk test, and homogeneity of variances was evaluated using Levene’s test. For single time-point comparisons between the two cultivars (JH vs. HJH), Student’s *t*-test was applied when both normality and equal variance assumptions were met; otherwise, the Mann–Whitney U test was used. For multiple comparisons involving the same gene or metabolite across four developmental stages (30, 60, 90, and 120 DAFB), the Benjamini–Hochberg false discovery rate (FDR) correction was applied to control for type I errors. Adjusted *p*-values (q-values) < 0.05 were considered statistically significant, and q < 0.01 highly significant. All statistical analyses were performed using SPSS version 18 (IBM, Chicago, IL, USA) and R software (version 4.2, with the stats and ggplot2 packages).

## 5. Conclusions

This study systematically elucidated the molecular mechanism underlying the peel color difference between the JH apple and its red bud sport strain HJH using a combination of physiological and biochemical assays, metabolomics, transcriptomics, and molecular biology approaches. The results demonstrate that the deep red peel phenotype of HJH apples is primarily attributable to the substantial accumulation of anthocyanins, rather than differences in chlorophyll content. Metabolomic analysis further revealed that cyanidin derivatives, particularly cyanidin-3-O-(6″-O-malonyl) glucoside, significantly accumulate in HJH peel, serving as the key material basis for its coloration. Transcriptomic and qRT-PCR analyses showed that the structural gene *MdDFR* in the anthocyanin biosynthesis pathway was consistently highly expressed during key developmental stages of HJH fruit, with its expression pattern highly consistent with the trend of anthocyanin accumulation, while most other structural genes did not exhibit stable differences. In conclusion, the upregulation of *MdDFR* is the core molecular event driving the specific accumulation of cyanidin-type anthocyanins in HJH peel, leading to its deep red coloration. This study provides new insights into the metabolic regulatory network underlying apple fruit color variation and offers important theoretical foundations and candidate genes for the genetic improvement of apple fruit color quality. Future research should focus on several directions: (1) functional validation of *MdDFR* through transient or stable genetic transformation to establish its causal role in the deep red phenotype; (2) identification and characterization of the specific malonyltransferase(s) responsible for the malonylation of cyanidin glycosides in apple, and elucidation of their transcriptional regulatory mechanisms; (3) exploration of upstream transcription factors (e.g., MYB, bHLH, WD40) that regulate *MdDFR* expression, thereby completing the regulatory network underlying the enhanced anthocyanin accumulation in HJH; and (4) investigation of the potential application of these findings in marker-assisted breeding for improved apple peel color.

## Figures and Tables

**Table 1:** Secondary classification of metabolites.

Primary Classification	Secondary Classification	Number
Flavonoids	Flavonols	137
	Flavones	87
	Dihydroflavones	31
	Flavanols	28
	Chalcones	22
	Tannins	15
	Proanthocyanidins	13
	Dihydroflavonols	10
	Anthocyanins	9
	Other flavonoids	7
	Isoflavones	3

**Table 2:** Differential metabolites enriched in KEGG pathways.

Enrichment Pathway	Number	Differential Metabolite	VIP	Log_2_FC	Fold Change	Regulation
Biosynthesis of secondary metabolites	10	5-Deoxy-5-methylthioadenosine	1.27	0.43	1.34	up
		1-Aminocyclopropane-1-carboxylic acid	1.42	0.47	1.39	up
		L-Malic acid	1.41	−0.38	0.77	down
		L-Serine	1.41	0.37	1.29	up
		Putrescine	1.34	0.53	1.45	up
		Xanthine	1.42	2.02	4.06	up
		Fumaric acid	1.43	−0.39	0.76	down
		Adenosine-5’-monophosphate	1.34	0.63	1.55	up
		L-Ornithine	1.43	0.60	1.51	up
		L-Histidine	1.37	0.95	1.93	up
Flavonoid and flavonol biosynthesis	2	Myricetin	1.25	0.65	1.57	up
		Acacetin	1.23	−0.47	0.72	down
Flavonoid biosynthesis	4	Leucocyanidin	1.36	1.33	2.52	up
		Myricetin	1.25	0.65	1.57	up
		Gallocatechin	1.34	0.62	1.53	up
		Epigallocatechin	1.42	0.77	1.70	up
Anthocyanin biosynthesis	3	Cyanidin-3-O-(2″-O-xylosyl) galactoside	1.43	1.71	3.26	up
		Cyanidin-3-O-glucoside	1.41	1.31	2.49	up
		Cyanidin-3-O-(6″-O-malonyl) glucoside	1.41	2.83	7.11	up

**Table 3:** Evaluation of sequencing data quality.

Samples	Clean Reads	Clean Bases	GC Content	% ≥ Q30
JH1	20,602,273	6,168,437,214	47.07%	93.29%
JH2	19,404,833	5,810,680,536	46.89%	93.65%
JH3	19,079,921	5,710,370,466	47.00%	93.50%
HJH1	21,242,001	6,359,952,312	46.95%	92.51%
HJH2	19,777,778	5,921,586,314	47.04%	93.37%
HJH3	20,687,332	6,195,256,536	46.90%	93.62%

Note: (1) Samples: sample identifiers; (2) clean reads: the number of paired-end reads in the clean data; (3) clean bases: the total number of bases in the clean data; (4) GC content: the proportion of guanine (G) and cytosine (C) bases in the clean data; (5) percentage of bases with Q-score ≥ 30 (%): the proportion of bases with a quality score of 30 or higher in the clean data.

**Table 4:** Differential structural genes in the anthocyanin biosynthesis pathway.

Gene Name	Gene ID	log_2_FC (HJH vs. JH)	Annotation
*MdDFR*	HF13503	1.15	hypothetical protein DVH24_012918 [Malus domestica]
*MdCHS*	HF00720	0.89	polyketide synthase 5 [Malus domestica]
*MdUFGT*	HF23295	1.32	UDP glucose: flavonoid 3-O-glucosyl transferase [Malus domestica]
*MdF3’H*	HF44548	−0.67	flavonol synthase/flavanone 3-hydroxylase-like [Malus domestica]
*MdLAR*	HF10027	−1.08	hypothetical protein DVH24_009592 [Malus domestica]

## Data Availability

Transcriptome data generated during this study have been submitted to the BioProject database at NCBI under the BioProject ID: PRJNA1447421. Restrictions apply to the availability of metabolomics data. Data were obtained from Biomarker Technologies Co., Ltd. (Beijing, China) and are available from the authors with the permission of the company.

## Supplementary Materials

The following supporting information can be downloaded at: https://www.mdpi.com/article/10.3390/ijms27177954/s1.
